# Sutures versus staples for wound closure in orthopaedic surgery: a randomized controlled trial

**DOI:** 10.1186/1471-2474-13-89

**Published:** 2012-06-06

**Authors:** Jesse A Shantz, James Vernon, Jeff Leiter, Saam Morshed, Gregory Stranges

**Affiliations:** 1 Pan Am Clinic, 75 Poseidon Bay, Winnipeg, MB, R3M 3E4, Canada; 2 Orthopaedic Trauma Institute at San Francisco General Hospital, University of California, San Francisco, 2550 23rd Street, Building 9, 2nd Floor, San Francisco, CA, 94110, USA; 3 Section of Orthopaedics, Dunedin School of Medicine, PO Box 913, Dunedin, New Zealand; 4 Section of Orthopaedic Surgery, University of Manitoba, AD-401, 820 Sherbrook St., Winnipeg, MB, R3A 1R9, Canada

**Keywords:** Wound closure, Orthopaedic surgery, Sutures, Staples, Surgical wound complications, Surgical site infection

## Abstract

**Background:**

A recently published meta-analysis comparing metallic staples to sutures in orthopaedic procedures revealed three fold increase in risk for infection in stapled wounds. The studies included in the meta-analysis are at risk of bias due to experimental design limitations. A large randomized controlled trial is proposed to direct orthopaedic surgeons in their choice of wound closure material.

**Methods/Design:**

A parallel group randomized controlled trial with institutional review board approval will be conducted. Patients will be randomized intraoperatively to have skin wounds closed with sutures or staples. Dressings will be used to maintain blinding outcome assessors. The primary outcome measure will be a composite all-cause wound complication outcome measure composed of: infection, wound drainage, wound necrosis, blistering, dehiscence, suture abscess and material sensitivity reaction. An independent review board blinded to treatment assignment will adjudicate suspected complications based on clinical data. All deceased patients will also be reviewed. An interim analysis of complications will take place after half of the patients have been recruited. All data will be analyzed by a blinded statistician. Dichotomous primary and secondary outcome measures will be analyzed using the Chi-squared statistic. Continuous outcome measures will be analyzed using Student's t-test. Subgroup analysis will compare infection rates using sutures versus staples in each anatomic area (upper extremity, pelvis/acetabulum, hip/femur, knee, ankle). A further subgroup analysis will be conducted comparing trauma patients to elective surgery patients. Non-infected revision surgery will also be compared to primary surgery.

**Discussion:**

Wound closure material is an afterthought for many orthopaedic surgeons. The combined results of several comparative trials suggests that the choice of wound closure materials may have an impact on the rate of surgical site infections. However, the strength of the evidence is poor given the heterogeneity of the methods employed in previous studies. The following study protocol aims to guide surgeons in their choice of wound closure material by determining if there is a difference in complication rates in sutured and stapled wounds.

**Trial Registration:**

This trial was registered at ClinicalTrials.gov under the identifier NCT01146236 (registered June 14, 2010)

## Background

### Surgical site infections

The Centers for Disease Control (CDC) defines surgical site infections (SSIs) as superficial incisional, deep incisional and organ/space infections based on clinical and laboratory confirmation [1]. Two-hundred and ninety-thousand SSIs occur in the United States annually following elective orthopaedic surgery resulting in $1 billion to $10 billion in additional healthcare costs according to CDC estimates from 2001. Many of these infections require long-term intravenous antibiotic treatment or further surgical interventions. It has been estimated that SSIs lead to a $4,500 per case (in 1990 dollars) increase in health care costs in the United States [2]. Some estimates have found that surgical site infections in hip and knee replacement can incur an additional cost in excess of $28,000 [3].

### Skin closure efficacy

Animal studies have shown the mechanical equivalence of stapled and sutured wounds [4]. Clinical studies in several specialties have not shown superiority of the cosmetic appearance of stapled or sutured wounds [5,6]. These authors also found that patient satisfaction with the two methods of wound closure was not significantly different. In practice, it is uncommon for a patient to receive the choice of closure method pre-operatively and it is also rare to find patients with absolute preferences to one closure material over another.

### Skin closure cost-effectiveness

Several studies have mentioned the time saved in inserting and removing staples when compared with sutures [5,7]. These studies rely on the calculation of the time saved by employing metallic staples compared to the cost of the staple insertion device. Singh et al. (2006) have previously published that staples are more than eight times more costly to insert and remove than are sutures [8]. No study has factored the cost of complications into a cost analysis comparing sutures and metallic staples.

No comparative trial exists that has the statistical power to determine if the rate of infection in wounds closed with staples is different from that in wounds closed with sutures in orthopaedic procedures. A study in cardiac procedures suggested that subcuticular suturing in sternal wounds led to a lower overall rate of complications than stapling [9]. Although the results of a recent meta-analysis [10] suggest that the use of staples in hip and knee surgery should be questioned, the included studies [7,8,11-14] did not uniformly employ experimental designs that limit bias. There is also a paucity of data to guide surgeons working in various anatomic locations on the type of wound closure material that confers the lowest risk of infection. A large, randomized and blinded prospective trial is needed to provide an answer to the question of what material is best for the closure of clean surgical wounds.

### Research question

In adult patients undergoing orthopaedic procedures does the wound closure material (sutures versus metallic staples) influence the occurrence of post-operative wound complications within the first year?

### Objectives

• To determine the best surgical skin wound closure material for use in various orthopaedic surgical procedures from a patient safety perspective.

• To identify procedures in which staple or suture closure results in an increased risk of wound complications.

• To determine if surgical wounds in trauma patients require different closure material than elective surgical wounds at distinct anatomic sites.

### Hypotheses

It is hypothesized that wounds closed with sutures and staples will have similar all-cause complication rates. The alternative hypothesis is that these wound closure methods result in different rates of complications indicating that one method is superior to the other and should be utilized for the tested procedures.

## Methods

### Study approval

This protocol was approved by the local institutional review board in May 2010. Participation is purely voluntary and withdrawal from the study after randomization will be allowed. The study was also registered at clinicaltrials.gov after institutional review board approval. Registration identification *NCT01146236* (*registered June 14, 2010)*

### Study sample

The study will be executed in five medical centers affiliated with two accredited universities. Patients will be drawn from a catchment of 2.7 million inhabitants. The study population will be a mix of rural and urban patients from diverse ethnic backgrounds. A variety of procedures will be included in the study encompassing both urgent and elective operations.

### Trial design

#### Parallel Group Randomized Controlled Trial with Economic Analysis

**Figure 1:**
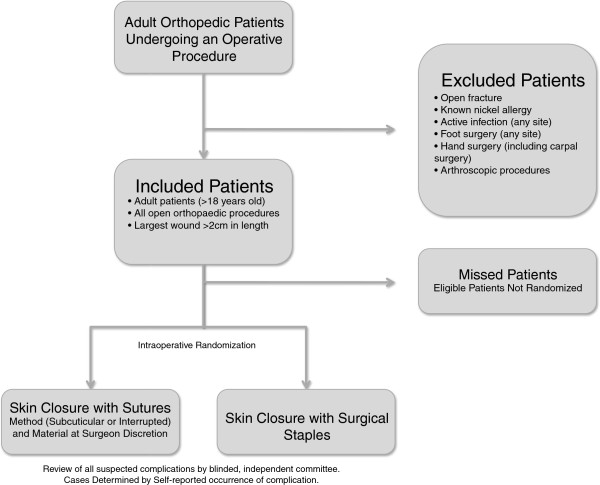
Participant flow diagram representing the planned design of the study.

Recruitment of patients under the care of subspecialists in Orthopaedic Trauma, Spine, Adult Reconstruction and Upper Extremity Reconstruction will take place concurrently with the same trial infrastructure. Patients will be recruited by the office assistant, research assistant or delegate of the treating surgeon administering the surgical consent form. Verbal consent will be obtained for trauma patients unable to sign a consent form. Written consent will be obtained within 24 hours of treatment allocation. Patients eligible but not enrolled will be documented. Participant flow through the study can be seen visually in Figure 1.

### Inclusion criteria

• Adult patients (> 18 years old)

• All open orthopaedic procedures

• Any wound > 2 cm in length

### Exclusion criteria

• Open fracture

• Known nickel allergy

• Active infection (any site)

• Foot surgery (any site)

• Hand surgery (including carpal surgery)

• Arthroscopic procedures

### Interventions

• Skin closure with metallic staples

• Skin closure with suture material – type of suture (non-absorbable or absorbable) and technique (simple, horizontal mattress, vertical mattress, subcuticular) at the discretion of the surgeon.

### Method of allocation to groups

Randomization will be concealed and will be allocated at the time of skin closure. Randomization will be completed using an online randomization program. Block randomization with randomly sized blocks between eight and 12 participants will be employed to ensure an equal number of participants in each group for each subspecialty. Group assignments will be revealed by the online randomization program and the time of the randomization will be recorded electronically. Times of randomization will be compared with operative notes retrospectively to ensure concealment is being maintained.

### Methods of blinding

Blinding of patients and providers was attempted in a pilot study. It was found that it was not feasible to blind these groups to the treatment allocation after post-operative dressings were changed, generally at postoperative day two. Additionally, post-operative radiographs revealed stapled patients in the pilot, further unblinding both providers and participants.

Outcome assessors will be blinded to the closure method through the use of long sleeved shirts, pants or a sleeve provided for the patient to wear. Blinding will be maintained by trained clinic staff aware of the purpose and methods of blinding. Participants will be asked not to reveal randomization when they become aware of treatment allocation during the course of the follow-up.

The outcomes adjudication committee will be blinded to treatment group during the determination of complications of individual patients. The data analysis team will also be blinded to the treatment groups during the synthesis of the results which will be presented as Group A and Group B in the draft manuscript.

### Primary outcome measure

The primary outcome measure will be a composite outcome encompassing all causes of wound complication. The clinical relevance of the primary outcome measure stems from the fact that the components of the composite outcome all represent occurrences that are patient important. The components consist of the following events:

1. Surgical site infection as defined by:

• Use of intravenous antibiotics

• Use of oral antibiotics

• Re-operation at same site

2. Wound drainage occurring after post-operative day two requiring a dressing change.

3. Wound Necrosis defined as blackening of the skin edges at the incision site or skin slough observed by providers or the participant.

4. Suture Abscess defined as the expulsion of deep suture material and purulent material without surrounding erythema.

5. Peri-incision Blistering defined as blistering at the edge of the incision along the entire length. Blistering at dressing tape site will be excluded from this definition, however, blistering due to wound tape application which is contiguous with the wound edge will be considered an event.

6. Wound Dehiscence as defined by the loss of apposition of the skin edges visible to the eye along the length of the incision.

7. Material Sensitivity as defined by a local reaction to metal or suture material resulting in skin changes along the entire incision length.

The primary outcome measure will be assessed during admission by review of the patient chart by site coordinators who will be blinded to the treatment allocation. Outcome assessors will be uniformly trained in the definitions of the components of the composite outcome score. Outpatient complications will be recorded through self-report questionnaires administered by site coordinators. All deceased patients will also be reviewed for occurrence wound complications. Final determination of events will be made by an independent outcomes adjudication committee based on blinded clinical data identified by the committee as necessary for the determination of complications. A sample of non-infected participants will also be reviewed by the committee as a negative control.

### Secondary outcome measures

#### Treatment preference reported by the participant

Additional unscheduled episodes of care as defined by: 

• Dressing changes by homecare/patient at home or self-reported visits to other healthcare professionals.

• Length-of-stay – Based on admission and discharge dates

• Visual analogue pain score for suture/staple removal

### Justification of the length of follow-up

The occurrence of SSIs happens primarily in the first three months post-operatively. The Centers for Disease Control (CDC) defines the timing of SSI as within the first 30 days unless a foreign body is implanted. In the presence of an implanted foreign body the surveillance period is extended to one year. Since the majority of orthopaedic surgeries result in the implantation of a metallic body we have extended the follow-up period to one year. Given the fact that the primary outcome measure is a composite measure related to the healing of a wound we will analyze the data after all participants have completed six-week follow-up. A phone survey will be performed at one year in order to conform with the CDC definition of SSI.

### Sample size determination

**Figure 2:**
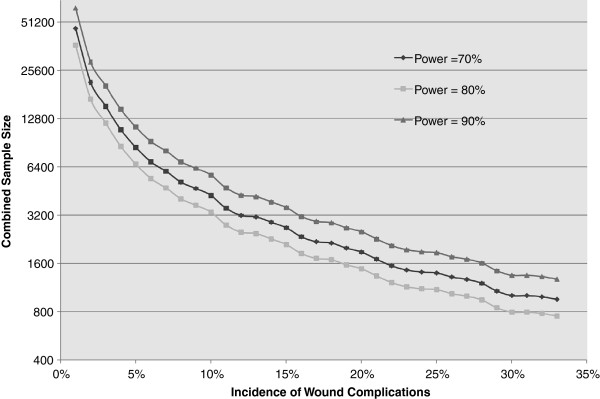
Projected sample size based on a relative risk reduction of 25% for wound complications and using an alpha of 0.05.

Estimates of sample size necessary to definitively test the hypothesis in this study were calculated using rates of wound complications found in a pilot study which included 148 participants. A relative risk reduction of 25% in wound complications was chosen as a minimal clinically important difference in wound complications. Sample sizes are presented graphically for varying powers and with a constant α level of 0.05 (Figure 2). Microsoft Excel (Redmond, WA) was utilized to perform calculations. The details of the sample size calculation are presented in Figure 2.

### Anticipated recruitment rate

In a pilot site it was found that 60% of patients approached consented to participate. A recruitment model was created taking into account expected site fatigue and differences in consenting efficiency between the United States and Canada. Based on the combined clinical volume at the five sites it is expected that the study will be completed with enrolment after 24 months of recruitment. Additional international sites are currently being developed to ensure that this target is met and to improve the generalizability of the results.

### Data analysis

All anatomic sites will be analyzed together and separately using Stata (College Station, TX). Dichotomous primary and secondary outcome measures will be analyzed using Fisher’s exact test. Stratification based on potential confounding variables will be performed. A multivariable analysis employing logistic regression will be performed to assess the possible impact of empirical confounding by putative risk factors of the binary outcomes. Length of stay, visual analogue scale pain measurements and patient satisfaction data will be compared using a two-way ANOVA. Linear regression will be performed on continuous variables to assess the possible impact of empirical confounding by putative risk factors. A 4.7% level of significance will be considered significant based on the adjustment of significance level for the interim analysis.

Subgroup analyses will be performed comparing the use of sutures and staples in trauma versus elective procedures and primary versus revision procedures as appropriate.

### Interim analysis

Interim analysis will be performed by a Data Safety and Monitoring Committee blinded to the treatment groups. Analyses will take place at the half-way point of patient recruitment. The alpha spending function approach to setting significance levels will be used. The trial will be stopped if a difference is found in the complication rates using a significance level of 0.15% based on the O’Brien-Flemming boundaries for a group sequential trial.

## Discussion

### Patient-centered outcomes

The importance of patient-centered outcomes is becoming more recognized in medicine. In orthopaedic surgery, the increasing popularity of patient-driven rather than surgeon-driven functional outcomes reflects this general trend. Little information exists on the incidence of patient-perceived wound complications in orthopaedic surgery. Based on the bias encountered in surgeon-measured outcomes there is a possibility that the current literature reflects an underestimate of the frequency of wound complications as a result of current definitions of these events and standard ways of determining their occurrence. This study additionally aims to find a true rate of patient-perceived wound complications to allow surgeons to better inform patients of surgical risks.

### Clinical relevance

The inclusion of both community and tertiary centers will help to make the results of this trial immediately applicable for surgeons in diverse areas of practice. Given the inclusion of both trauma and elective patients and as a result of the powering of the study for each anatomic location, the results will also inform practitioners if there is a difference in the rate of complications as a result of wound closure material at those individual sites. The results will reflect the realities of practice in both a teaching and community environment.

## Competing interests

The authors declare that they have no competing interests.

## Authors’ contributions

All listed authors participated in the design of the above trial. Drs. J.V. and J.S. prepared the manuscript and literature review. All authors read and approved the final manuscript.

## Ethics

All ethical considerations were approved by the University of Manitoba Biomedical Research Ethics Board.

## Funding

Initial funding for this study has been provided by the Manitoba Institute for Patient Safety through the Dr. John Wade Research Award, the Manitoba Medical Services Foundation, the Department of Surgery at the University of Manitoba, the AO North America Foundation through a resident research grant and the Section of Orthopaedic Surgery at the University of Manitoba through the Alexander Gibson Fund.

## Pre-publication history

The pre-publication history for this paper can be accessed here:

http://www.biomedcentral.com/1471-2474/13/89/prepub
